# Tadpole Skin Secretions, Not Food or Temperature, Mediate Costly Cannibal‐Induced Plasticity in Invasive Cane Toad Hatchlings

**DOI:** 10.1002/ece3.71094

**Published:** 2025-03-30

**Authors:** Michael R. Crossland, Richard Shine, Jayna L. DeVore

**Affiliations:** ^1^ School of Life and Environmental Sciences A08 The University of Sydney Sydney New South Wales Australia; ^2^ Department of Natural Sciences Macquarie University Sydney New South Wales Australia; ^3^ ILM, Ifremer, IRD, UMR 241 SECOPOL University of French Polynesia Faa'a Tahiti French Polynesia

**Keywords:** anura, *Bufo marinus*, developmental acceleration, irreversible cost, trade‐off

## Abstract

Hatchlings of invasive cane toads ( *Rhinella marina* ) in Australia respond facultatively to chemical cues of non‐feeding cannibalistic conspecific tadpoles by accelerating development, but consequently experience reduced growth, development and survival in the subsequent tadpole stage. Predation‐induced developmental acceleration of eggs or hatchlings is rare among amphibians, and the implications of and context‐dependent impacts of such developmental plasticity are poorly understood. For cane toads, the source and identity of the tadpole cue that induces this response are unknown. Additionally, it is unknown whether these carry‐over costs are due to accelerated early development per se or are specific to developmental acceleration induced by conspecific tadpole cues. Finally, it is unknown whether these costs can be mitigated by the availability of food resources at critical times during early development. We conducted laboratory experiments to investigate these issues. Our results show that (1) based on significant hatchling responses to skin swabs, the chemical that induces costly developmental plasticity is located in the skin of cannibalistic cane toad tadpoles, (2) carry‐over effects of early developmental acceleration are elicited only by cues from cannibal tadpoles because temperature‐induced developmental acceleration of hatchlings did not reduce subsequent growth, development or survival and (3) excess food availability during early development did not mitigate the carry‐over costs of exposure to cannibal tadpole cues. Thus, this developmental plasticity response, triggered by detection of chemicals exuded from the skin of conspecific tadpoles, causes unique negative carry‐over costs for younger larvae. However, we found that tadpole production of skin secretions is also plastic, with swabbed tadpoles inducing stronger responses in hatchlings than their unswabbed siblings. Finally, the carry‐over costs that follow cannibal exposure cannot be mitigated by favorable nutritional conditions.

## Graphical abstract

Hatchlings of invasive cane toads respond to the risk of cannibalism by accelerating development but experience significant negative carry‐over effects on fitness in the tadpole stage. Our experiments demonstrate that this hatchling response is induced by chemicals located in the skin of older cannibalistic tadpoles. This response is unique to cannibal tadpole cues, and once initiated, cannot be reversed by favourable conditions such as a high nutrient environment.

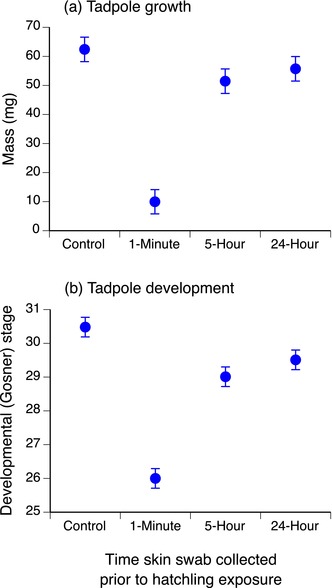

## 1 Introduction

The ability of an organism to adjust its development rate and/or trajectory in response to environmental conditions during ontogeny, known as developmental plasticity, is phylogenetically widespread (Benard 2004; Beldade et al. 2011; Taborsky 2017; Warkentin 2011a). In particular, the early life stages of anuran amphibians exhibit substantial developmental plasticity in response to external cues that predict threat or opportunity, such as the risk of predation or desiccation (Warkentin 1999, 2011a, 2011b; Gomez‐Mestre et al. 2006; Van Buskirk 2016; Schulte et al. 2023). The logistics of working with anuran amphibians are enhanced by their wide geographic distribution, high abundance, large clutch sizes and ease of maintenance of eggs, hatchlings and tadpoles in small experimental containers and larger mesocosms. As a result, anurans have become model organisms for research on the ways in which unpredictable environmental events can induce rapid shifts in developmental parameters such as the time of hatching (Van Buskirk 2016), larval morphology (Van Buskirk and McCollum 2000; Relyea 2001, 2004; Schulte et al. 2023), and the time to metamorphosis (Wilbur and Collins 1973; Laurila and Kujasalo 1999; Relyea 2007; Székely et al. 2017). That research has revealed a diverse array of apparently adaptive responses, some induced by chemical or physical predation stimuli (Warkentin 1999; Van Buskirk 2016; Schulte et al. 2023) and others involving broader responses to overall conditions of temperature, food supply, and competitor density (Relyea 2001; Doughty and Roberts 2003; Touchon and Warkentin 2011).

Recent research has revealed an unusual type of developmental plasticity in the cane toad ( *Rhinella marina* ). In the species' invasive range in Australia, but not within its native range source population in French Guiana, toad tadpoles are voracious consumers of newly laid conspecific eggs and hatchlings (DeVore et al. 2021a). The hatchling stage, when hatchlings have emerged from the egg capsule but are not yet capable of swimming or feeding, is when the risk of cannibalism is most pronounced (Crossland et al. 2022). Since cane toads hatch relatively early in development by dissolving the egg capsule (~Gosner 1960 stage 17), these hatchlings are relatively immobile, but cannibalistic cane toad tadpoles present in the pond can detect their maternally invested bufadienolide toxins and are actively attracted to this vulnerable life stage (DeVore et al. 2021a, 2021b; Crossland et al. 2022). In response to this risk, when exposed to chemical cues from conspecific tadpoles, toads in most Australian clutches accelerate development during the late hatchling stage to more quickly reach the free‐swimming tadpole stage that is not vulnerable to cannibalism (DeVore et al. 2021b). Such predator‐induced developmental acceleration during the pre‐feeding egg or hatchling stages is unusual in amphibians, having only been shown in two other species (Kishida et al. 2015; Segev et al. 2015); a more typical response to predation risk has been found in amphibians that respond to such cues by altering egg hatching time without altering development rate (Van Buskirk 2016; Crossland et al. 2024). For example, Gomez‐Mestre et al. (2006) found that American toads typically do not hatch until Gosner stage 19, but hatch ~2 days early at Gosner stage 17 in response to infection by water mould. In contrast, cane toads inherently hatch upon reaching hatching competence at ~Gosner stage 17 and have not been found to alter the timing of hatching in response to cannibal cues; apparently, accelerated development is their only defense against cannibalism (Crossland et al. 2024).

For cane toads in Australia, the cannibal‐induced increase in hatchling development rate occurs as a trade‐off with reduced hatchling growth (Crossland et al. 2024) and confers a high subsequent cost: hatchlings that accelerate development when exposed to non‐feeding cannibal tadpole cues subsequently grow and develop more slowly and survive less well as tadpoles (Crossland and Shine 2012; Clarke et al. 2015, 2016; McCann et al. 2019a, 2020; DeVore et al. 2021a, 2021b; Crossland et al. 2024). The magnitude of cannibal‐induced developmental acceleration correlates with the magnitude of subsequent costs: clutches with rapid cannibal‐induced developmental acceleration show lower subsequent viability (DeVore et al. 2021b) with the cannibal‐induced size reduction at the hatchling/tadpole transition point predicting future survival (Crossland et al. 2024). Experimental studies show that the cannibal cue that induces developmental plasticity in cane toad hatchlings in Australia: (1) is produced by free‐swimming cane toad tadpoles from Gosner stage 25 onwards but not by earlier developmental stages, (2) is produced by cane toad tadpoles regardless of the presence or absence of conspecific eggs and hatchlings, (3) is produced by non‐feeding cane toad tadpoles, (4) is not a micro‐organism associated with toad tadpoles but is likely a chemical produced by toad tadpoles themselves, although not an ammonia‐based chemical, and (5) is species‐specific: cane toad tadpole cues do not affect native frog hatchlings, and native frog tadpole cues do not affect cane toad hatchlings (Clarke et al. 2015, 2016; our unpubl. data).

However, several aspects of the cane toad cannibalism system in Australia remain unresolved. Firstly, the source and identity of the cue have yet to be identified. One possibility is that the cue is located in the skin of cane toad tadpoles. Amphibian skin contains a variety of chemicals that, if released into the surrounding environment, might be used by hatchlings to identify potential cannibals (Demori et al. 2019). For example, damage to the skin tissue of toads ( *Rhinella arenarum* ) releases an epidermal peptide that causes alarm responses in conspecifics (Raices et al. 2020; Jungblut et al. 2022), while tetrodotoxin located in the skin of cannibalistic adult California newts ( *Taricha torosa* ) induces strong anti‐predator avoidance behaviour in conspecific larvae (Zimmer et al. 2006). In cane toads, the chemical(s) responsible for cannibal‐induced developmental plasticity are not alarm chemicals because effects are induced by exposure to healthy, undamaged conspecific tadpoles. In this sense, the cane toad response is similar to that documented in the California newt: for both species, the chemical is non‐facultatively exuded by free‐ranging, undamaged cannibal individuals into the aquatic environment and induces a response in conspecific prey irrespective of the feeding status of the cannibal individual (Zimmer et al. 2006; Crossland et al. 2024).

Secondly, it is unknown whether accelerated development during hatchling stages is inherently costly to cane toads. In our work on this system (Crossland and Shine 2012; Clarke et al. 2015, 2016; McCann et al. 2019a, 2020; DeVore et al. 2021a, 2021b; Crossland et al. 2024), we have interpreted the carry‐over costs of exposure to cannibal cues as a specific response to those cues. For cane toads, accelerated development during hatchling stages occurs as a trade‐off with hatchling growth, such that the consequent size reduction at the completion of the hatchling stage predicts future survival (Crossland et al. 2024). However, another possibility is that rapid embryogenesis, in general, incurs negative carry‐over effects. Developmental rate in anurans can be affected by other factors including water temperature (Mitchell and Seymour 2000; Sanuy et al. 2008), and thermal conditions during embryogenesis can influence the size and morphology of larval amphibians (Jonsson et al. 2022) as well as other aquatic organisms (e.g., fish: Jonsson and Jonsson 2014). Does acceleration of development caused by other factors, such as higher temperatures, induce the same kinds of carry‐over effects as does cannibal‐induced acceleration? Answering that question can clarify the costs of cannibal‐induced developmental plasticity in larval cane toads.

In addition, nutritional conditions during early development can have dramatic effects on life‐history trajectories for many organisms (Metcalfe and Monaghan 2001), including anurans (Audo et al. 1995; Álvarez and Nicieza 2002; Ramamonjisoa et al. 2016). For the cane toad cannibalism system in Australia, all laboratory experiments conducted to date have followed the same protocol: eggs and hatchlings were raised in a nutrient‐free environment, and feeding only commenced when the non‐feeding hatchlings had transitioned into feeding, free‐swimming Gosner stage 25 tadpoles (Crossland and Shine 2012; Clarke et al. 2015, 2016; McCann et al. 2019a, 2020; DeVore et al. 2021a, 2021b; Crossland et al. 2024). This methodology could potentially influence the extent of previously documented carry‐over effects in several ways. Firstly, the highly permeable skin or external gills of toad hatchlings or early‐stage tadpoles may enable them to supplement their nutrition by absorbing nutrients directly from the surrounding water. If so, the low‐nutrient conditions during early development in previous experiments may have exacerbated the carry‐over effects of cannibal‐induced developmental plasticity. We are unaware of any studies demonstrating that anuran hatchlings or tadpoles have such capacity to absorb aqueous nutrients through the skin, but other aquatic taxa do have this ability (e.g., hagfish: Glover et al. 2013). In addition, some amphibians may absorb nutrients through their external gills (e.g., pouch brooding marsupial frogs; Warne and Catenazzi 2016). Secondly, even if cane toad hatchlings are unable to absorb nutrients from the surrounding environment, the mere presence of waterborne cues of such nutrients can potentially alter growth trajectories. For example, when reared with waterborne cues of fairy shrimp nauplii, non‐feeding hatchlings of the Mexican spadefoot toad ( *Spea multiplicata* ) develop increased body size and jaw muscle size—traits that increase the ability of spadefoot toad tadpoles to prey upon these shrimp at later tadpole developmental stages (Harmon et al. 2023). Thirdly, it is unknown whether developmental acceleration could allow hatchlings to begin feeding at an earlier developmental stage, reducing their otherwise complete reliance on endogenous resources prior to stage 25. Finally, the difference between the time when hatchlings exhaust yolk sac reserves as they transform into stage 25 tadpoles and the time experimenters in previous studies noticed this transition had occurred and commenced feeding of tadpoles may mean that early‐stage tadpoles had no access to any food for several hours. This absence of food at the moment yolk reserves are exhausted could potentially exacerbate the costs of the growth/development trade‐off during the hatchling stage, especially if endogenous resource exhaustion occurs earlier in individuals exposed to cannibal cues (DeVore et al. 2021b; Crossland et al. 2024).

We therefore conducted a series of laboratory experiments to investigate the following: (1) whether the cue that induces negative carry‐over effects of cannibal‐induced developmental plasticity is located in the skin of cane toad tadpoles, (2) whether temperature‐induced accelerated development of cane toad hatchlings causes negative carry‐over effects comparable to those induced by cannibal‐induced developmental acceleration, and (3) whether food availability during hatchling and early‐stage tadpole development mitigates the carry‐over costs of cannibal‐induced developmental plasticity in cane toads.

## 2 Materials and Methods

Cane toads ( *Rhinella marina* ; formerly *Bufo marinus* ) are large bufonid anurans native to South America and translocated to Australia in 1935 for control of insect pests (Shine 2018). Adults produce large clutches to > 40,000 eggs (DeVore et al. 2021b) that are laid in temporary and permanent water bodies. Eggs hatch relatively early in development at Gosner stage 17–18, ~48 h after egg deposition, and hatchlings develop into free‐swimming feeding tadpoles (stage 25) another 24–48 h later. Here we consider the period between when the eggs hatch and the beginning of the feeding stage 25 tadpole as the ‘hatchling stage’. However, note that the developmental stage at which hatching occurs can vary between both species and individuals, though anurans typically hatch between Gosner stages 17–20 (Gosner 1960). Cane toad tadpoles are omnivorous, principally grazing on algae, but in Australia, they are highly cannibalistic on conspecific eggs and hatchlings. Metamorphosis can occur within 2–3 weeks depending on density and water temperature (see Crossland et al. 2024 for summary).

We collected adult cane toads from several localities throughout their range in Australia (Queensland: Innisfail, Townsville; Northern Territory: Middle Point; Western Australia: Ellenbrae, Kununurra, Oombulgurri, Palm Creek) and housed them at the Tropical Ecology Research Facility, Middle Point, Northern Territory (12°34′43.54″ S, 131°18′51.55″ E) in outdoor bins (1 m × 1 m × 0.8 m) with refugia, water and a constant food supply.

We induced adult toads to spawn by subcutaneous injection of 0.25 mg mL^−1^ synthetic gonadotrophin leuprorelin acetate (Lucrin, Abbot Australasia). Male toads were injected with 0.25 mL, and female toads with 0.75 mL. Pairs of toads were placed in covered 80 L plastic tubs with a small amount of water and allowed to spawn overnight. The following morning, eggs were removed and placed in 18 L plastic tubs filled with 9 L water and constantly aerated. Eggs and hatchlings were haphazardly selected from these tubs for use in experiments, as required. Hatchlings were added to experimental containers without any remnants of the egg string in case the latter served as a food source during the exposure period. Hatchlings not used as focal individuals in experiments were left to develop into mobile, feeding stage 25 tadpoles (Gosner 1960), at which time they were transferred to shaded outdoor, screen‐covered 750 L bins. These tadpoles were fed daily (Hikari Algae Wafers, Kyorin, Japan) with weekly water changes and haphazardly selected to generate cannibal cues in experiments, as required. Using this protocol, tadpoles used to generate cannibal cues were from different, older clutches than responding egg clutches, as occurs in nature. All experiments were conducted in an indoor laboratory (25°C–27°C) using non‐chlorinated well water. Cane toad tadpoles used to generate cues were not fed during experiments for consistency with all our previous experiments and because feeding is not required for cane toad tadpoles to induce developmental plasticity in conspecific hatchlings (see Crossland et al. 2024 for summary). All tadpole developmental stages below refer to Gosner (1960).

### 2.1 Experiment 1. Is the Chemical Cue Located in Tadpole Skin?

We exposed hatchlings from a single Middle Point egg clutch to skin swabs from a single tadpole clutch (Kununurra; stage 30–38 tadpoles). We collected tadpole skin swabs by gently pressing a cotton bud (Baby Cotton Buds, Johnsons) against the body and tail of tadpoles. We swabbed three tadpoles per cotton bud, rotating the bud between swabs to ensure a clean section was used for each tadpole. Tadpole swabs were collected at intervals of 1 min, 5 h and 24 h prior to exposure to hatchlings. Our aim was to determine whether skin swabs induce carry‐over developmental responses in hatchlings, and if so, whether the swabbed chemical is stable over a 24 h period. After collection, all swabs plus control swabs were placed on a tray on the laboratory benchtop until used in the experiment.

We added groups of 10 hatchlings (stages 18 and 19) to 75 mL plastic containers holding 50 mL water. Hatchlings were randomly allocated to one of four swab treatments, using one cotton bud per container: (1) blank swab (control), (2) skin swab collected 1 min previously, (3) skin swab collected 5 h previously, or (4) skin swab collected 24 h previously. Each treatment was replicated five times. When we noticed hatchlings had developed into mobile, feeding stage 25 tadpoles (~72 h later), the cotton buds were removed and five tadpoles per container were haphazardly chosen and placed in 1 L plastic containers holding 750 mL fresh water, located on the laboratory benchtop. Since tadpole growth can be sensitive to even minor variation in light or temperature, containers were arranged in 2 × 2 spatial blocks, such that each of the five blocks contained one replicate per treatment. The position of each tank was randomized within each block. This spatial blocking design was used across all experiments. These tadpoles were fed powdered algae wafers (Kyorin, Japan) *ad libitum* for 10 days, with water changed every 3 days. After 10 days, the number of surviving tadpoles in each container was recorded. Tadpoles were then euthanized using MS‐222 (tricaine methanesulfonate) and the body mass (blotted dry weight, mg) and developmental stage of each tadpole were measured.

### 2.2 Experiment 2. Does Swabbing of Tadpoles Alter Their Potency to Induce Carry‐Over Effects?

We also tested whether the act of tadpoles being swabbed alters their potency to induce carry‐over effects in conspecific hatchlings, using the same tadpole and egg clutches as in Experiment 1. Ten hatchlings (stage 18–19) were added to 1 L plastic containers holding 750 mL water, placed on the laboratory benchtop. Hatchlings were randomly allocated to one of three treatments: (1) control, (2) exposure to three live tadpoles swabbed 5 h previously that were the live swabbed tadpoles from treatment 3 of Experiment 1 above or (3) exposure to three live un‐swabbed tadpoles that were full siblings of the tadpoles in treatment 2 in this experiment. Each treatment was replicated five times. The live tadpoles were placed in a fibreglass net (1 mm^2^ mesh) suspended above developing hatchlings to allow exposure to tadpole cues while preventing predation on hatchlings. Chemical cues could reach the hatchlings through the mesh both via diffusion and via water flow caused by tadpole swimming activity. Visual cues such as tadpole silhouettes could have been perceived by the hatchlings, but only once hatchlings reached Gosner stage 21, at which point the cornea becomes transparent (Gosner 1960). Control tubs had a mesh net without tadpoles.

When hatchlings became mobile at stages 23 and 24 (~48 h later) and were at risk of swimming through the mesh net and being eaten, we removed the live tadpoles but kept hatchlings in the cue water. A further 24 h later, when hatchlings had developed into stage 25 tadpoles, five individuals per container were haphazardly chosen and transferred to new 1 L containers holding 750 mL fresh water placed on the laboratory benchtop in a randomised block design. These tadpoles were raised for 10 days, at which time they were counted, measured and staged as described in Experiment 1.

### 2.3 Experiment 3. Is the Chemical Cue Also Found in Adult Parotoid Gland Secretions?

Experiment 1 demonstrated that the carry‐over effects for hatchlings exposed to non‐feeding cane toad tadpoles are induced by chemicals located in the skin of these tadpoles (see Section 4). Given that exposure to native Australian frog tadpoles does not induce such carry‐over effects for cane toad hatchlings (our unpubl. data), the cue must be a chemical or chemicals present in the skin of cane toad tadpoles but absent from the skin of native frog tadpoles. This precludes any generic tadpole skin chemicals that are common among anuran taxa.

As a first step in identifying potential chemical(s) responsible for negative effects induced by cannibal toad tadpoles, we tested the response of toad hatchlings to secretions from the parotoid glands of adult cane toads. We did this because our previous studies on the chemistry of cannibalism attraction showed that adult cane toad parotoid secretions elicit the same attraction response by cane toad tadpoles as do the chemical cues from hatchlings that have just emerged from the egg string (Crossland et al. 2012; DeVore et al. 2021a, 2021b). Subsequent work demonstrated it was bufadienolide chemicals, present in both adult parotoid secretions and embryo stages, that cause this attraction response (Crossland et al. 2021). Bufadienolides are potential candidates for the observed cannibal‐induced effects on hatchlings because these chemicals are present in the skin of bufonid tadpoles (including cane toad tadpoles: Flier et al. 1980, Jara and Perotti 2009) but absent from the skin of native Australian frog tadpoles (there are no native bufonid species in Australia). However, the secretions of adult bufonid toad parotoid glands contain not only bufadienolides but also a variety of other compounds, including peptides, biogenic amines and alkaloids (Chen and Kovaríková 1967; Toledo and Jared 1995; Clarke 1997; Alexandre et al. 2021; Kowalski et al. 2023). Thus, any negative effect of adult cane toad parotoid secretions on developing hatchlings would indicate the chemical(s) responsible are present in both parotoid secretions and tadpole skin, but would not identify which specific chemical(s) are involved.

To test whether parotoid gland secretions induce an anti‐cannibal response in cane toad hatchlings, we squeezed 600 mg of parotoid secretion from 20 adult toads (Middle Point) by hand into 30 mL of water to create a 20 mg/mL parotoid secretion solution. The solution was left at laboratory room temperature for 30 min before being added to experimental containers (as per attractant experiments of McCann et al. 2019b). We placed 10 hatchlings (stage 18) from a single Middle Point clutch into 1 L plastic containers holding 750 mL of water. Hatchlings were randomly allocated to one of five treatments: (1) Control, (2) 1 mL of parotoid secretion solution added to containers at the start of the experiment ( = 0.027 mg/mL parotoid secretion in containers), (3) 5 mL of parotoid secretion solution added to containers at the start of the experiment ( = 0.133 mg/mL parotoid secretion in containers), (4) cues from three live tadpoles (one clutch: Innisfail) or (5) cues from three live tadpoles (one clutch: Kununurra). Live tadpoles were suspended above developing hatchlings via a fibreglass mesh net (1 mm^2^ mesh) while the control had a mesh net without tadpoles, as per Experiment 2. The 0.027 mg/mL parotoid secretion treatment was chosen because this approximates the concentration of adult parotoid secretion known to elicit attraction in cane toad tadpoles (McCann et al. 2019b: 0.020 mg/mL adult secretion). The 0.133 mg/mL parotoid secretion treatment was chosen to assess concentration effects of exposure to parotoid secretion. Live tadpole treatments were included for comparison with the parotoid secretion treatments.

Each parotoid secretion treatment was replicated three times; all other treatments were replicated five times. For cannibal‐exposed treatments, the cannibal tadpoles were removed when hatchlings became mobile after 48 h, but we retained the hatchlings in the cannibal tadpole source water. When hatchlings reached stage 25, five individuals were haphazardly selected from each container and transferred to new 1 L containers holding 750 mL fresh water, placed in a randomised block design. These tadpoles were raised for 10 days before being counted, measured and staged as described in Experiment 1.

### 2.4 Experiment 4. Is Developmental Acceleration Inherently Costly for Cane Toad Hatchlings?

We allocated groups of 10 eggs from a single Middle Point clutch to 75 mL plastic containers holding 50 mL water. These containers were first randomly allocated to one of two acceleration treatments: temperature vs. cannibal tadpole cues. Within each of these groups, containers were subsequently randomly allocated to treatment (temperature‐induced acceleration: low vs. high temperature; cannibal‐induced acceleration: control vs. cannibal tadpole cues). Responding eggs were stage 10–11 at the commencement of treatment exposure. We started treatment exposure in this experiment at an earlier developmental stage than our other experiments to maximise the potential for temperature effects on the rate of embryogenesis. Earlier exposure to cannibal tadpole cues in this experiment was done to ensure results were directly comparable to the temperature treatment. We note that earlier exposure to cannibal tadpole cues was unlikely to produce carry‐over results different from our other experiments, which started exposure at the hatchling stage 17–18: Clarke et al. (2015) exposed cane toad eggs and hatchlings at varying Gosner development stages to tadpole cannibal cues (exposure during stage 10–18, exposure during stage 18–21, exposure during stage 21–25, exposure during all stages 0–25) and found no difference among treatments in carry‐over effects on tadpole growth and survival.

For temperature‐induced developmental acceleration, we placed 5 replicate 75 mL containers in each of two incubators (Incufridge). We recorded temperature within each incubator at 12 h after the start of temperature exposure using a hand‐held InfraRed thermometer (Digitech QM7215), and thereafter every 6 h until all individuals within the incubator had developed into stage 25 tadpoles. The low‐temperature incubator (mean = 25.5°C, SD = 0.6°C, range = 24.3°C–26.6°C) was set to levels comparable to ambient air temperature in the laboratory (25°C–27°C). The high temperature incubator was set to ~9°C higher than the low temperature incubator (mean = 34.0°C, SD = 0.4°C, range = 33.3°C–34.9°C). This temperature range is within that documented in cane toad breeding pools (17°C–36.6°C) and approximates local breeding pool temperatures at Middle Point (mean: 32°C; Wijethunga et al. 2016; Hagman and Shine 2006, Ducatez, Shine and DeVore unpubl. data). Additionally, our aim in using a difference of ~9°C between temperature treatments was to ensure a strong difference in embryonic development rate, thus allowing us to evaluate any carry‐over costs of temperature‐induced developmental acceleration during embryogenesis. Development time for each individual was calculated as the time from placement in incubators at stage 10–11 until stage 25.

For cannibal‐induced developmental acceleration, eggs were transferred from the initial 75 mL containers to 1 L containers holding 750 mL water. We used 4 clutches to generate tadpole cues (1 Innisfail, 1 Oombulgurri, 2 Kununurra) with five replicates for the control treatment and each tadpole clutch. The live tadpoles were placed in a fibreglass net (1 mm^2^ mesh) suspended above developing hatchlings as described in the experiments above. Control tubs had a mesh net without tadpoles. We did not directly measure the time of development to stage 25 in the cannibal‐exposed treatments. However, we have previously demonstrated that any negative carry‐over effects on subsequent tadpole growth, development or survival for cannibal‐exposed toad hatchlings, as demonstrated in this experiment (see Section 4), are the result of the acceleration of hatchling development rate (DeVore et al. 2021b).

For all containers in temperature and cannibal tadpole developmental acceleration treatments, when hatchlings transitioned into stage 25 tadpoles, we haphazardly chose five tadpoles per container and transferred these to new 1 L containers holding 750 mL fresh water. These 1 L containers were placed in a randomised block design on a laboratory benchtop and raised for 10 days, at which time we recorded mass, stage and survival as described above.

We have previously demonstrated that cannibal‐induced developmental acceleration occurs due to a growth/development trade‐off during late hatchling stages, resulting in significantly reduced mass of stage 25 tadpoles (DeVore et al. 2021b; Crossland et al. 2024). To assess whether such a growth/development trade‐off also occurs for hatchlings exposed to temperature‐induced developmental acceleration, we measured the mass (blotted dry weight, mg) of stage 25 tadpoles in low and high temperature containers for excess individuals not chosen for the carry‐over 10 days growth experiment.

### 2.5 Experiment 5. Does Early Access to Food Resources Offset the Costs of Cannibal‐Induced Developmental Plasticity?

We conducted two experiments to assess the effect of food subsidies on the costs of cannibal‐induced developmental plasticity.

### 2.6 Experiment 5.1

We tested hatchlings from a single Middle Point clutch in a fully crossed design of absence (−) or presence (+) of food subsidy (F) and cannibal cues (C) using four treatments: (1) F−C−, (2) F−C+, (3) F+C− and (4) F+C+.

We generated the food subsidy (F) treatments by filling twenty 1 L plastic containers with 750 mL of water. Half the containers were randomly chosen to be F+ treatments, and we added 50 mg of powdered Hikari algae wafers (Kyorin, Japan) to these containers. The remaining containers had no algae wafers added (F− treatments). We placed all containers outside in direct sunlight for 48 h to generate a bio‐community in the F+ containers and then moved all containers into the laboratory to start the experiment. At the completion of this 48 h sunlight exposure period, organic material could be seen with the naked eye in the F+ containers, whereas the water in the F‐ containers remained clear.

We then randomly allocated the cannibal cue treatment (C−, C+) to all food treatment containers. We generated cannibal tadpole cues using five tadpole clutches (one clutch each of Townsville, Ellenbrae, Palm Creek; two clutches Innisfail). Each of the five tadpole clutches was set up with one replicate of each of the four food subsidy/cannibal cue treatments listed above. We then randomly allocated 15 eggs (stage 11) to each container. For the cannibal‐cue treatments, we generated cues by placing 3 tadpoles in a fibreglass net (1 mm^2^ mesh) suspended above the developing eggs as described in the experiments above. Within 8 h of hatching, we removed empty egg strands to ensure that they were not available as a food source. When hatchlings were stages 23 and 24, cannibal tadpoles were removed, but hatchlings remained in their original cue water. Control containers had a suspended mesh with no live tadpoles.

All containers were checked at 6 h intervals throughout the day and night. When we noticed hatchlings had reached stage 25, we haphazardly split the tadpoles within each rearing container into two replicate groups of five tadpoles and then placed each group of five tadpoles in a new 1 L container with fresh 750 mL water and fed them immediately. This replication was achieved for all treatment combinations with the exception of three combinations where low survival meant we could only set up one replicate of stage 25 tadpoles in fresh water (Ellenbrae F+C−, Ellenbrae F+C+, Innisfail F−C+). Tadpoles were raised for 10 days before being counted, weighed and staged, as described above.

This experimental design gave developing eggs and hatchlings in F+ containers the opportunity to absorb nutrients directly from the water, if that is possible. Additionally, it gave tadpoles in F+ containers access to external food the moment they exhausted the embryonic yolk sac and transformed into feeding stage 25 tadpoles. In contrast, eggs, hatchlings and tadpoles in F‐ containers had no access to external food until after we observed they had reached stage 25, which occurred sometime within the previous 6 h, at which time we fed them.

### 2.7 Experiment 5.2

We conducted a follow‐up experiment to Experiment 5.1 using enhanced food subsidies (algae wafers + natural pond dirt) and to further explore the interaction between the timing of access to food resources and the effects of cannibal tadpole cues on the costs of developmental plasticity. This experiment used the same initial fully crossed design of food subsidy × cannibal cue as per Experiment 5.1, with the addition that once hatchlings transitioned to stage 25, the tadpoles within each rearing container were split into a further treatment of either fed immediately at stage 25 or fed 24 h after reaching stage 25. Thus, this experiment was a 3 factorial design: food (F)−/+, cannibal cue (C)−/+ and time stage 25 tadpoles fed (immediately/24 h later).

We established the F+ treatments by adding 75 mL dry sediment collected from a local temporary pond used by cane toads for breeding and 50 mg algae wafer powder (Kyorin, Japan) to twelve 1 L plastic containers filled with 750 mL water. Another 12 containers used for F− treatments had no sediment or algae wafer powder added. All containers were placed outside in direct sunlight for 5 days to allow the bio‐community to develop in the F+ containers before being returned indoors to the laboratory at the start of the experiment. As in Experiment 5.1, organic matter was visually evident in the F+ containers at the completion of the sunlight exposure period (in this case, 5 days) but was absent in F− containers.

We randomly allocated 20 eggs (stage 11) from a single Middle Point clutch to food subsidy (F) and cannibal cue (C) treatments as per Experiment 5.1. Cannibal cues were derived using six tadpole clutches (one clutch each of Townsville, Oombulgurri, Ellenbrae, Kununurra; two clutches Innisfail). Each of the six tadpole clutches was set up with a single replicate of the four initial F/C crossed treatments: (1) F–C−, (2) F–C+, (3) F+C− and (4) F+C+. Exposure to cannibal tadpole cues was as per Experiment 5.1.

All containers were checked at 6 h intervals throughout the day and night. When we noticed hatchlings had reached stage 25, we haphazardly chose two groups of five tadpoles within each container and placed each group in a new 1 L container with 750 mL fresh water. One container was randomly chosen to feed tadpoles immediately as per Experiment 5.1, whereas tadpoles in the other container were not fed until 24 h later. We followed this design for all tadpole clutches with one exception: the container for Ellenbrae F+ C+ FedLate was omitted due to insufficient numbers of surviving tadpoles. Tadpoles were grown for 10 days, after which they were counted, measured, and staged as described above.

## 3 Statistical Analyses

We conducted all analyses in R (R Core Team 2023). Growth and development data were analysed using linear mixed effect models (package nlme:lme; Pinheiro et al. 2023). Survival data were analysed as a binomial response (alive, dead) using logistic regression (Warton and Hui 2011) and quasi‐binomial models to account for overdispersion (package MASS:glmmPQL, Ripley et al. 2023) followed by Anova (package car: Fox et al. 2019). Significant overall effects involving more than two treatments were followed by post hoc Tukey's tests (package multcomp; Hothorn et al. 2023).

We initially included density as a covariate in the growth and development models to account for potential survival‐related effects on these responses among treatments. These models included all interaction terms between density and each main effect (Yzerbyt et al. 2004), with density centred on the median number of surviving tadpoles at the end of the experiment. When centred density *p* was < 0.1, we retained it as a covariate in the model; otherwise, we removed it and re‐ran the model. Further details for specific models are listed below.

### 3.1 Experiments 1 and 2. Tadpole Skin Swab Analyses

For tadpole skin swab analyses and swabbed tadpole analyses, the tank was nested within the spatial block as a random effect for growth and development models, while the spatial block was used as a random effect for survival models. Because the cannibal tadpole cue and the volume of water used during hatchling cue exposure differed between skin swab and swabbed tadpole experiments, which may have influenced cue potency, we analysed the results of each experiment separately.

### 3.2 Experiment 3. Is the Chemical Cue Also Found in Adult Parotoid Gland Secretions?

We combined data for the live Innisfail and Kununurra tadpole treatments into a single estimate for the effect of exposure to live cannibal tadpole cues. For growth and development models, the tank was nested within the spatial block as a random effect. For the survival model, the spatial block was used as a random effect. Because survival was 100% in the 5 mL parotoid secretion treatment, the survival model could not reach convergence. To overcome this issue, we assigned one tadpole of this treatment to have died to allow a conservative estimate of effect for day 10 survival (Warton and Hui 2011).

### 3.3 Experiment 4. Temperature Versus Cannibal Tadpole Cue Analyses

We tested for interaction effects of development rate (slow, fast) × accelerated development cue (temperature, cannibal tadpole cue) on tadpole mass, stage and survival. For these analyses, the tank was nested within the spatial block as a random effect for growth and development models, while the spatial block was used as a random effect in survival models. For models assessing the development rate × accelerated development cue interaction that included density as a covariate, and for the model assessing the specific effect of cannibal cue treatment on survival, the models could not reach convergence because there was 100% survival in three of the four treatments (low temperature, high temperature and cannibal control). Consequently, we allocated a single tadpole to have died in each of these treatments to obtain a conservative estimate of effect (Warton and Hui 2011). We did not formally assess treatment effects on survival for temperature‐induced accelerated development as a main effect because all tadpoles in the low and high treatment containers survived to day 10.

### 3.4 Experiment 5. Does Early Access to Food Resources Offset the Costs of Cannibal‐Induced Developmental Plasticity?

We first assessed whether the time at which stage 25 tadpoles were fed in Experiment 5.2 (fed immediately vs. 24 h later) affected growth, development or survival, using models with growth tub nested within exposure tub and cannibal tadpole clutch as random effects. The survival model could not reach convergence due to very high survival in the F−C− and F+C− treatments. Therefore, we allocated one tadpole in each of these treatments to have died to obtain a conservative estimate of treatment effect on day 10 survival (Warton and Hui 2011). The time that stage 25 tadpoles were fed did not affect day 10 tadpole mass (treatment *F* = 0.75, df = 1,19, *p* = 0.40; density *p* < 0.0001), development stage (treatment *F* = 0.70, df = 1,19, *p* = 0.41; density *p* < 0.0001) or survival (*χ* ^2^ = 0.25, df = 1, *p* = 0.62). Therefore, we disregarded this treatment as a factor and combined the data for Experiments 5.1 and 5.2 in subsequent analyses.

We then assessed whether food subsidy type (algae wafer powder [Experiment 5.1] vs. algae wafer powder + pond dirt [Experiment 5.2]) affected growth, development or survival, using the combined data for both experiments with models including growth tub nested within exposure tub and cannibal tadpole clutch as random effects. Food subsidy type affected day 10 tadpole mass (treatment *F* = 14.63, df = 1,34, *p* < 0.0001; density *p* < 0.0001) and development stage (treatment *F* = 22.60, df = 1,34, *p* < 0.0001; density *p* < 0.0001) but had no significant effect on day 10 survival (*χ* ^2^ = 0.69, df = 1, *p* = 0.41). Overall, hatchlings raised in tubs seeded with algae wafer powder only were larger in mass and stage at day 10 than those raised in tubs seeded with algae wafer powder + pond dirt (mean mass [mg] ± SE = 58.59 ± 2.16 vs. 49.95 ± 1.85; mean stage = 32.19 ± 0.29 vs. 30.61 ± 0.24). There was no difference between the two food subsidy experiments in the control (F−C−) treatment (day 10 mass: *F* = 0.03, df = 1,3, *p* = 0.87; day 10 development stage: *F* = 1.66, df = 1,3, *p* = 0.29; day 10 survival: chi‐square = 0.02, df = 1, *p* = 0.89), indicating there was no inherent difference between the two clutches used for each food subsidy experiment in terms of growth, development or survival. That is, differences in effects of food subsidy type on day 10 mass and development stage were not confounded with a clutch effect for responding individuals.

Based on the above results for the effect of food subsidy type, we then tested for effects of food subsidy (no, yes) × cannibal tadpole cue (no, yes) using the combined dataset for both experiments with models for mass, development stage and survival, also including food subsidy type as a covariate. Growth tub was nested within exposure tub and cannibal tadpole clutch as random effects in each model. For each model, when food subsidy type *p* was < 0.1, we retained it as a covariate in the model; otherwise, we removed it and re‐ran the model. The survival model could not reach convergence because there was 100% survival in two treatments (F+C−, F−C−). To obtain a conservative estimate of effect for day 10 survival, we allocated one tadpole to have died in each of these two treatments (Warton and Hui 2011).

## 4 Results

### 4.1 Experiment 1. Is the Chemical Cue Located in Tadpole Skin?

There were significant overall treatment effects of hatchling exposure to tadpole skin swabs for tadpole growth (mass: *F* = 32.07, df = 3,12, *p* < 0.0001) and development (stage: *F* = 44.19, df = 3,12, *p* < 0.0001) at day 10, but no effect for day 10 survival (*χ* ^2^ = 0, df = 3, *p* = 1.0).

**FIGURE 1:**
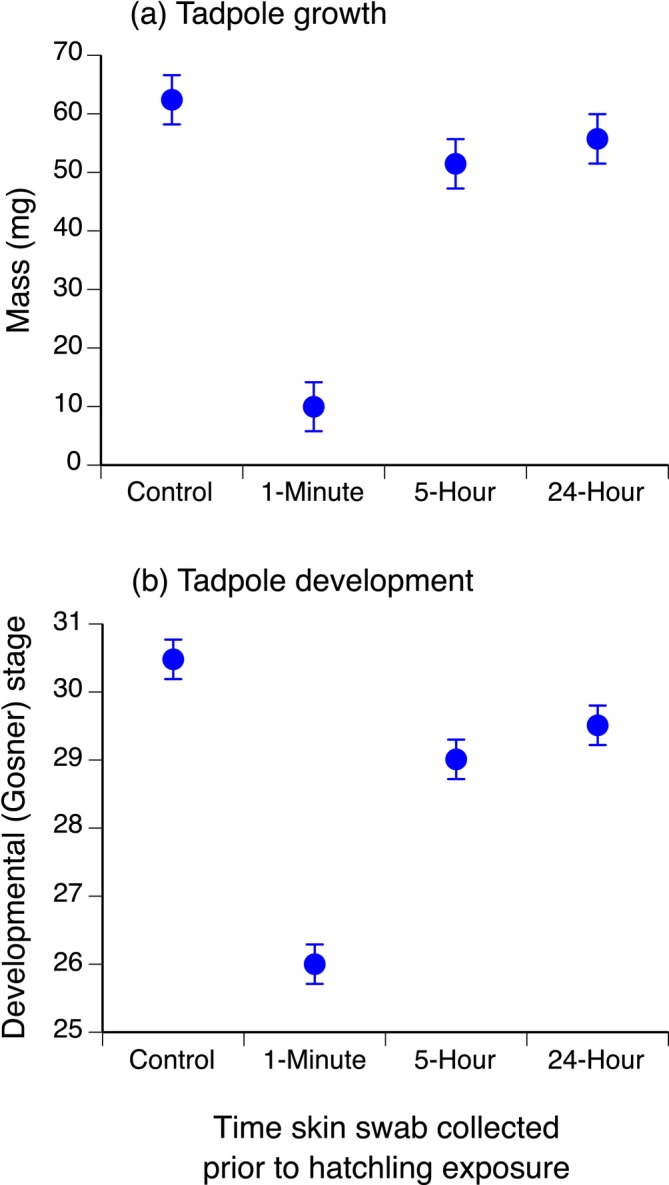
Effect of hatchling exposure to cannibal tadpole skin swabs on tadpole growth (mass [mg]), mean ± SE; (a) and development (Gosner 1960) stage, mean ± SE; (b) at day 10.

Skin swabs collected 1 min prior to exposure to toad hatchlings caused negative carry‐over effects on day 10 tadpole mass compared to all other treatments, while skin swabs collected 5 h and 24 h previously had no carry‐over effect on day 10 tadpole mass (Table 1, Figure 1a). Skin swabs collected at 1 min, 5 h, and 24 h caused negative carry‐over effects on development stage at day 10, with the strongest effects being for 1 min swabs (Table 1, Figure 1b). Negative effects of 5 h swabs and 24 h swabs on day 10 development were equivalent (Table 1, Figure 1b). Only 3 of 100 tadpoles died by day 10 (1 control, 1 tadpole exposed to 5 h skin swab and 1 tadpole exposed to 24 h skin swab).

**TABLE 1:** Multiple comparisons (Tukey contrasts) for the effect of hatchling exposure to toad tadpole skin swabs on growth (mass) and development (Gosner 1960 stage) at day 10 (See Figure 1).

Treatment comparison	Estimate ± SE	*z*	*p*
Tadpole mass (mg)			
1 min swab vs. control swab	−52.44 ± 5.94	−8.83	< 0.0001*
5 h swab vs. control swab	−10.94 ± 5.97	−1.83	0.200
24 h swab vs. control swab	−6.68 ± 5.97	−1.12	0.526
24 h swab vs. 1 min swab	45.76 ± 5.94	7.71	< 0.0001*
5 h swab vs. 1 min swab	41.50 ± 5.94	6.99	< 0.0001*
5 h swab vs. 24 h swab	−4.26 ± 5.97	−0.71	0.526
Tadpole development stage			
1 min swab vs. control swab	−4.48 ± 0.41	−10.88	< 0.0001*
5 h swab vs. control swab	−1.47 ± 0.41	−3.54	0.0012*
24 h swab vs. control swab	−0.97 ± 0.41	−2.35	0.0379*
24 h swab vs. 1 min swab	3.51 ± 0.41	8.52	< 0.0001*
5 h swab vs. 1 min swab	3.01 ± 0.41	7.32	< 0.0001*
5 h swab vs. 24 h swab	−0.50 ± 0.41	−1.20	0.2318

*p < 0.05.

### 4.2 Experiment 2. Does Swabbing of Tadpoles Alter Their Potency to Induce Carry‐Over Effects?

There were significant overall treatment effects at day 10 for tadpole mass (*F* = 63.66, df = 2,8, *p* < 0.0001), development stage (*F* = 226.75, df = 2,8, *p* < 0.0001) and survival (*χ* ^2^ = 24.29, df = 2, *p* < 0.0001).

**FIGURE 2:**
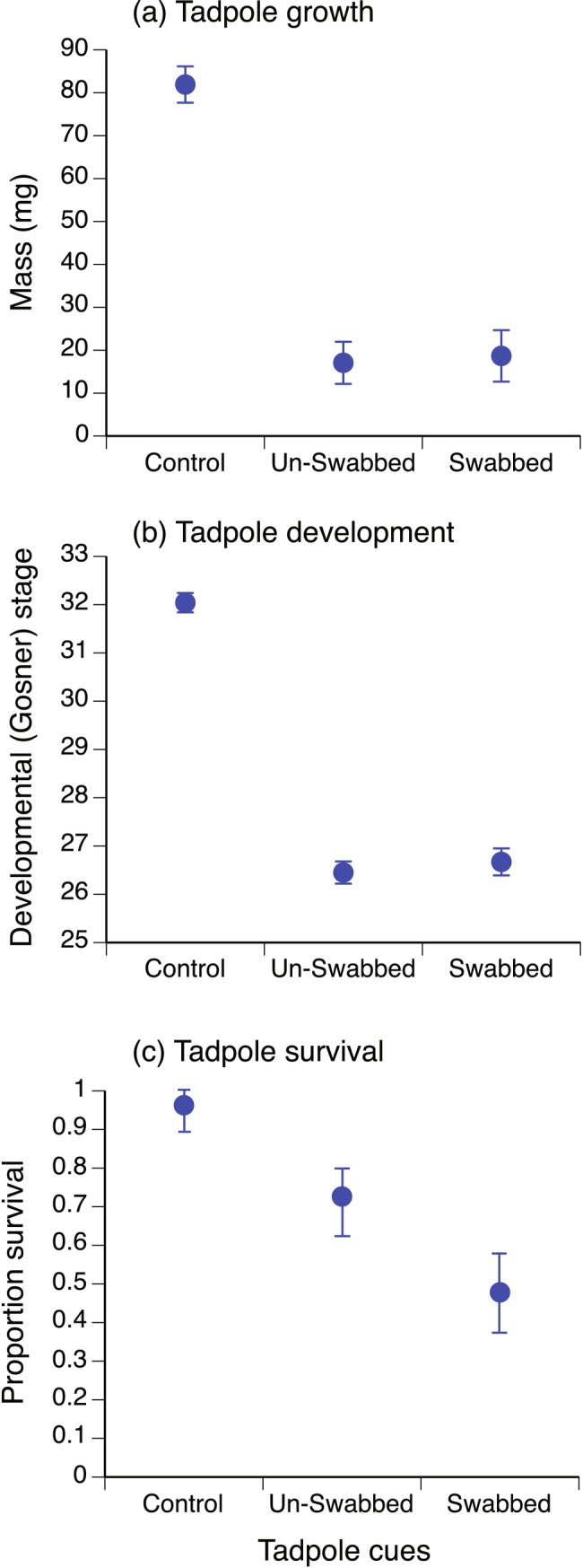
Effect of hatchling exposure to control conditions, cannibal tadpoles not previously swabbed, and cannibal tadpoles swabbed 5 h previously on tadpole growth (mass (mg), mean ± SE); (a), development (Gosner 1960 stage, mean ± SE); (b) and survival (proportion alive, mean ± SE); (c) at day 10.

Hatchling exposure to swabbed and un‐swabbed tadpoles caused equivalent negative carry‐over effects on growth and development at day 10 (Table 2, Figure 2a,b). However, cues from swabbed tadpoles caused a greater reduction in survival at day 10 than cues from un‐swabbed tadpoles (Table 2, Figure 2c). Control tadpoles were 9.3 times more likely to survive to day 10 than siblings exposed to un‐swabbed tadpole cues (odds ratio SE 3.2–27.1) but 26.0 times more likely to survive than siblings exposed to swabbed tadpole cues (odds ratio SE 9.5–71.07). Tadpoles exposed as hatchlings to un‐swabbed tadpole cues were 3.1 times more likely to survive to day 10 than siblings exposed to swabbed tadpole cues (odds ratio SE 2.7–3.7).

**TABLE 2:** Multiple comparisons (Tukey contrasts) for the effect of hatchling exposure to toad tadpole swab treatment (control, tadpole not swabbed, tadpole swabbed 5 h previously) on growth (mass), development (Gosner 1960 stage) and survival at day 10 (see Figure 2).

Treatment comparison	Estimate ± SE	*z*	*p*
Tadpole mass (mg)			
Tadpole not swabbed vs. control	−64.86 ± 6.48	−10.01	< 0.0001*
Tadpole swabbed vs. control	−63.25 ± 7.35	−8.61	< 0.0001*
Tadpole swabbed vs. tadpole not swabbed	1.61 ± 7.75	0.21	0.835
Tadpole development stage			
Tadpole not swabbed vs. control	−5.60 ± 0.29	−19.00	< 0.0001*
Tadpole swabbed vs. control	−5.38 ± 0.33	−16.10	< 0.0001*
Tadpole swabbed vs. tadpole not swabbed	0.22 ± 0.35	0.63	0.53
Tadpole survival			
Tadpole not swabbed vs. control	−2.28 ± 0.72	−3.15	0.0033*
Tadpole swabbed vs. control	−3.34 ± 0.71	−4.67	< 0.0001*
Tadpole swabbed vs. tadpole not swabbed	−1.06 ± 0.39	−2.69	0.0071*

*p < 0.05.

### 4.3 Experiment 3. Is the Chemical Cue Also Found in Adult Parotoid Gland Secretions?

There were significant overall treatment effects at day 10 for tadpole mass (*F* = 21.66, df = 3,13, *p* < 0.0001), development stage (F = 31.41, df = 3,13, *p* < 0.0001) and survival (*χ* ^2^ = 17.87, df = 3, *p* = 0.0005).

**FIGURE 3:**
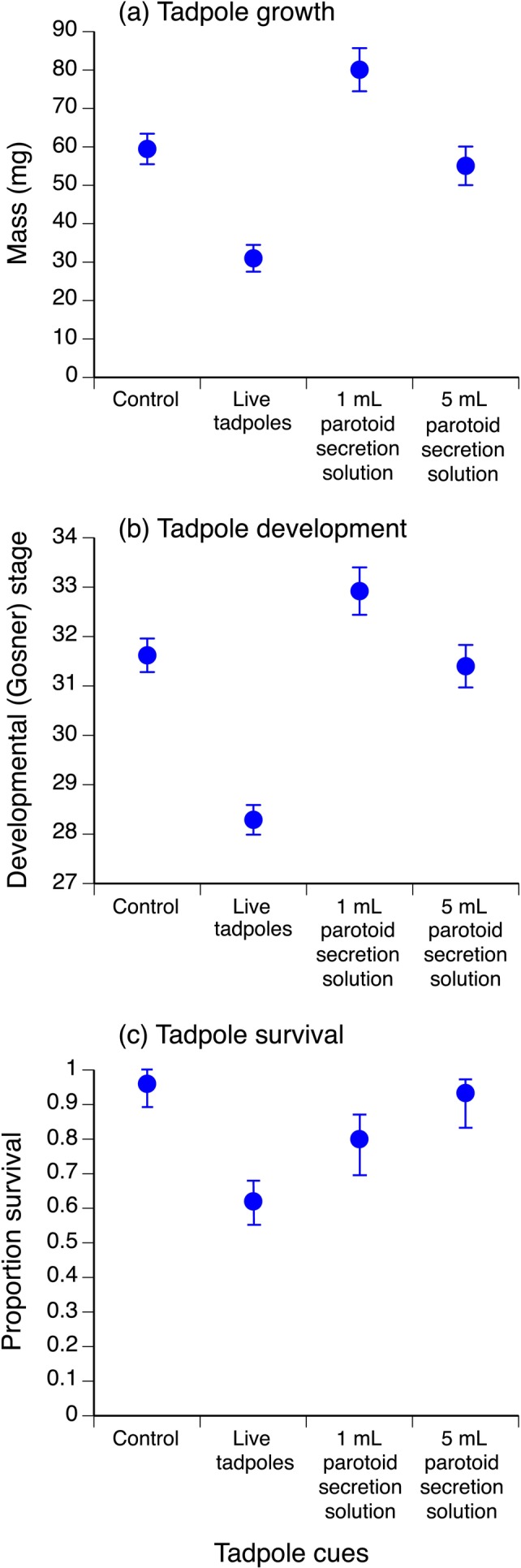
Effect of hatchling exposure to control conditions, cannibal tadpoles, 1 mL adult toad parotoid secretion solution, and 5 mL adult toad parotoid secretion solution on tadpole growth (mass [mg], mean ± SE); (a), development (Gosner 1960 stage, mean ± SE); (b) and survival (proportion alive, mean ± SE); (c) at day 10.

Relative to the control, hatchling exposure to cannibal tadpole cues resulted in reduced growth, development and survival at day 10 (Table 3, Figure 3a–c). In contrast, relative to the control, early exposure to 1 mL of secretions of adult toad parotoid glands resulted in significantly increased mass at day 10, with also a trend for increased development stage (*p* = 0.06) but no effect on survival (Table 3, Figure 3a–c). Exposure to 5 mL secretions of adult toad parotoid glands had no effect on growth, development or survival relative to the control at day 10 (Table 3, Figure 3a–c).

**TABLE 3:** Multiple comparisons (Tukey contrasts) for carry‐over effect of hatchling exposure to toad tadpole cues and adult cane toad parotoid gland secretion cues on growth (mass), development (Gosner 1960 stage) and survival at day 10 (see Figure 3).

Treatment comparison	Estimate ± SE	Z	*p*
Tadpole mass (mg)			
Live tadpoles vs. control	−28.46 ± 5.28	−5.39	< 0.0001*
1 mL parotoid vs. control	20.63 ± 6.87	3.00	0.0053*
5 mL parotoid vs. control	−4.39 ± 6.39	−0.69	0.4921
1 mL parotoid vs. live tadpoles	49.08 ± 6.60	7.43	< 0.0001*
5 mL parotoid vs. live tadpoles	24.07 ± 6.11	3.94	0.0003*
5 mL parotoid vs. 1 mL parotoid	−25.02 ± 7.52	−3.33	0.0026*
Tadpole development stage			
Live tadpoles vs. control	−3.33 ± 0.46	−7.33	< 0.0001*
1 mL parotoid vs. control	1.29 ± 0.59	2.18	0.0581
5 mL parotoid vs. control	−0.23 ± 0.55	−0.41	0.6830
1 mL parotoid vs. live tadpoles	4.63 ± 0.57	8.13	< 0.0001*
5 mL parotoid vs. live tadpoles	3.11 ± 0.53	5.91	< 0.0001*
5 mL parotoid vs. 1 mL parotoid	−1.52 ± 0.65	−2.34	0.0579
Tadpole survival			
Live tadpoles vs. control	−2.69 ± 0.81	−3.33	0.0053*
1 mL parotoid vs. control	−1.79 ± 0.92	−1.95	0.2051
5 mL parotoid vs. control	−0.54 ± 1.11	−0.49	0.6262
1 mL parotoid vs. live tadpoles	0.90 ± 0.54	1.66	0.2886
5 mL parotoid vs. live tadpoles	2.15 ± 0.82	2.63	0.0432*
5 mL parotoid vs. 1 mL parotoid	1.25 ± 0.93	1.35	0.3546

*Note:* ‘Parotoid’ refers to secretions obtained by squeezing the parotoid glands of adult toads by hand. Parotoid secretion is comprised of a mixture of compounds, including bufadienolides, peptides, biogenic amines and alkaloids (see Section 2 for details).

*p < 0.05.

### 4.4 Experiment 4. Is Developmental Acceleration Inherently Costly for Cane Toad Hatchlings?

**FIGURE 4:**
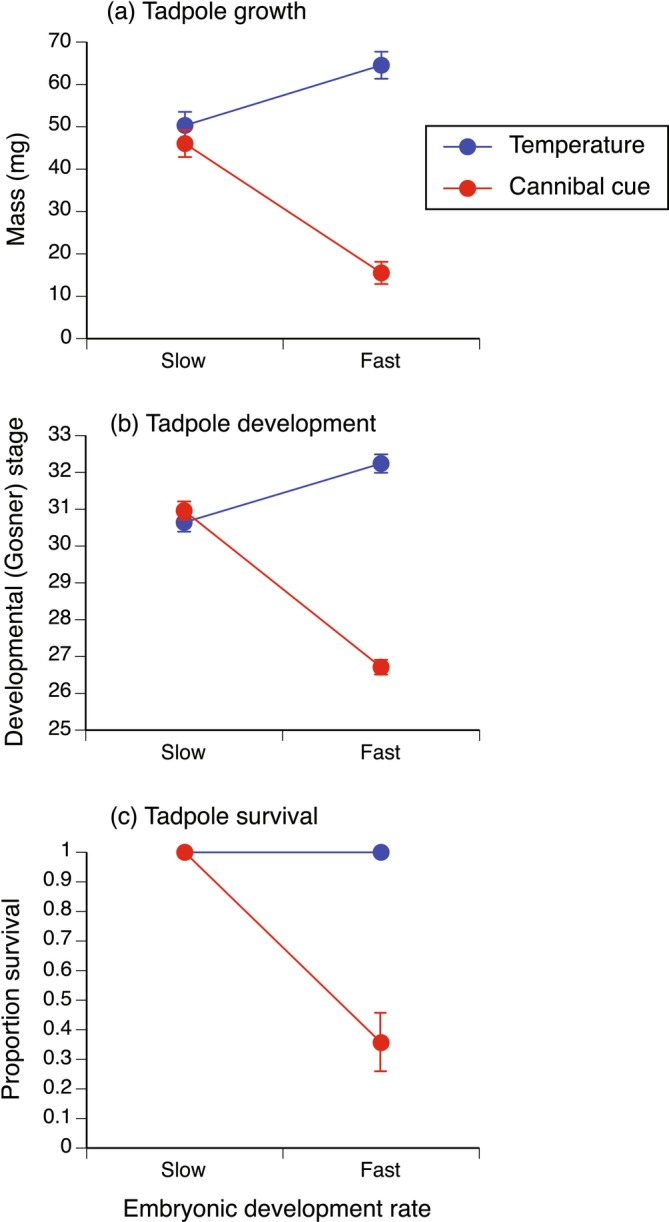
Effect of pre‐feeding development rate (slow, fast) via different development acceleration mechanisms (temperature, cannibal tadpole cues) on tadpole growth (mass (mg), mean ± SE); (a), development (Gosner 1960 stage, mean ± SE); (b) and survival (proportion alive, mean ± SE); (c) at day 10. For the temperature mechanism, slow development was induced by low temperature, while fast development was induced by high temperature. For the cannibal tadpole mechanism, slow development was induced by not exposing hatchlings to cannibal tadpole cues (i.e., control treatment) while fast development was induced by exposure to cannibal tadpole cues (see DeVore et al. 2021b for explanation of cannibal‐induced developmental acceleration).

As expected, increased temperature resulted in an increased development rate: the median development time to reach stage 25 in the high temperature treatment was approximately half that in the low temperature treatment (respectively, 48.5 h [range 48–49.5 h] vs. 90 h [range 89–90 h]). Exposure to cannibal tadpole cues also induced developmental acceleration of hatchlings, as evidenced by the significant negative carry‐over effects on growth, development and survival (Table 4, Figure 4a–c; also see DeVore et al. 2021b, Crossland et al. 2024 for interpretation of negative carry‐over effects).

**TABLE 4:** Effect of pre‐feeding developmental rate (slow, fast) and acceleration cue (temperature, cannibal tadpole cue) on growth (mass), development (Gosner 1960 stage) and survival of tadpoles at day 10 (see Figure 4).

Treatment	Estimate ± SE	df	*F* / *χ* ^2^	*p*
Tadpole mass (mg)				
Temperature (high temperature)	14.20 ± 5.37	1,4	6.99	0.0574
Cannibal tadpole cue (exposed)	−31.21 ± 3.34	1,17	87.11	< 0.0001*
Development rate × acceleration cue		1,22	38.61	< 0.0001*
Tadpole development stage				
Temperature (high temperature)	1.60 ± 0.30	1,4	28.28	0.006*
Cannibal tadpole cue (exposed)	−5.34 ± 0.56	1,16	213.67	< 0.0001*
Development rate × acceleration cue		1,22	124.97	< 0.0001*
Tadpole survival				
Temperature (high temperature)	—	—	—	—
Cannibal tadpole cue (exposed)	0.02 (0.01,0.05)	1	19.58	< 0.0001*
Development rate × acceleration cue		1	8.60	0.0034*

*Note:* Interaction results are from the full interaction model. Main effect results are from separate analyses for individual models for each factor. Density was retained as a covariate for the interaction model for tadpole mass (density *p* = 0.001), the interaction model for development stage (density *p* < 0.0001), and for the model assessing the main effect of cannibal cue on development stage (density *p* = 0.056); for all other models, density (*p* > 0.1) was removed as a covariate. *F* values are for mass and development stage results; chi‐squared value is for survival result. Survival was not analyzed as a main effect in the temperature treatment because survival was 100% in all low and high temperature treatment containers. Effect size (Estimate) for survival is odds ratio, with lower and upper SE values in parentheses.

*p < 0.05.

We have previously demonstrated that accelerated development of cane toad hatchlings in response to cannibal tadpole cues occurs at the expense of hatchling growth (Crossland et al. 2024), with a consequent significant reduction in tadpole mass at stage 25 relative to the control (*p* < 0.0001: DeVore et al. 2021b). In contrast, in the present study, there was no evidence of a growth/development trade‐off in the temperature experiment: stage 25 tadpoles in the low and high temperature treatments were similar in mass (*F* = 0.99, df = 1,8, *p* = 0.3479; mean mass [mg] ± SE: low temperature 6.32 ± 0.36 vs. high temperature 6.85 ± 0.39). That is, both increased temperature and exposure to cannibal tadpole cues accelerate development, but only the latter incurs a trade‐off with hatchling growth.

The interaction of development rate (slow vs. fast) × acceleration cue (temperature vs. cannibal cue) was significant for all response variables at day 10 (Table 4; Figure 4a–c), indicating that the two different mechanisms of accelerated development caused different carry‐over effects in the subsequent tadpole stage. Individual analyses of the main factors of acceleration cue showed that increased developmental rate via higher temperature resulted in a non‐significant trend for increased day 10 mass (*p* = 0.06), significantly increased day 10 development stage, and no effect on day 10 survival (Table 4, Figure 4a–c). In contrast, increased developmental rate via exposure to cannibal cues caused a significant reduction in day 10 mass, development stage, and survival (Table 4, Figure 4a–c). For the cannibal treatment, tadpoles in control containers were 49 times more likely to survive to day 10 than were tadpoles that had been exposed to cannibal tadpole cues as hatchlings (odds ratio SE: 19.6, 122.4).

### 4.5 Experiment 5. Does Early Access to Food Resources Offset the Costs of Cannibal‐Induced Developmental Plasticity?

**FIGURE 5:**
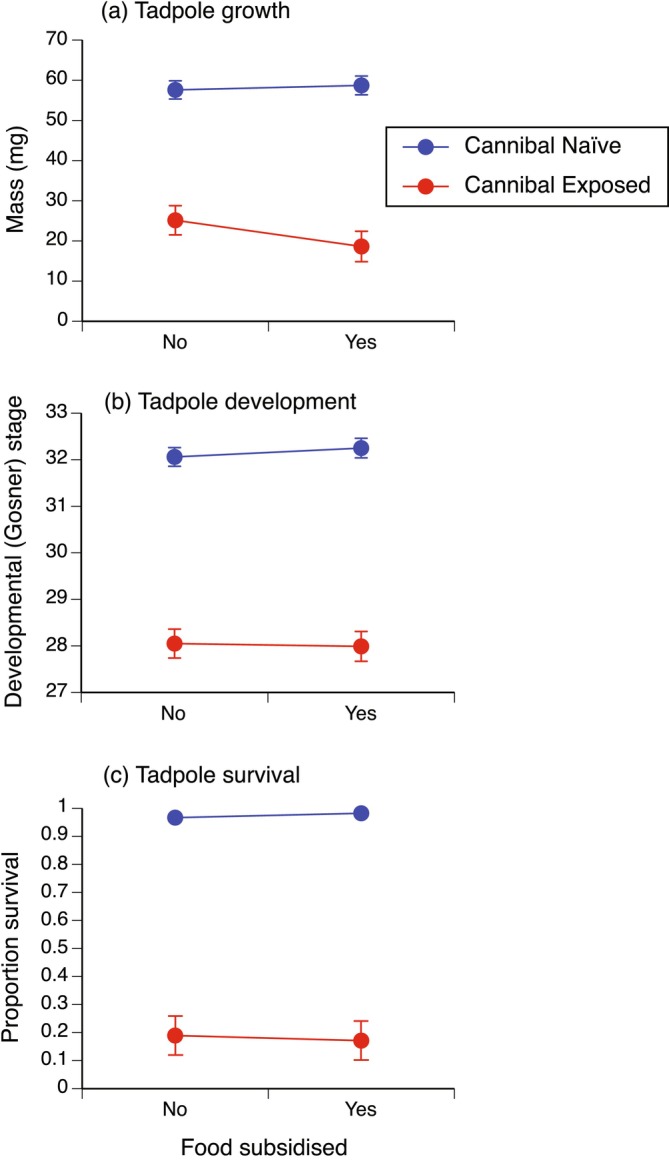
Effect of food subsidisation (no, yes) and exposure to cannibal tadpole cues (no = cannibal naïve, yes = cannibal exposed) during hatchling development on tadpole growth (mass (mg), mean ± SE); (a), development (Gosner 1960 stage, mean ± SE); (b) and survival (proportion alive, mean ± SE); (c) at day 10.

There was no significant interaction between food subsidy and cannibal tadpole cue for mass or survival at day 10 (respectively, *p* = 0.16, *p* = 0.97), but there was a significant (*p* = 0.04) interaction for day 10 development stage (Table 5; Figure 5a–c). Nonetheless, individual analyses of main effects showed a consistent pattern: food subsidy had no effect on mass, stage or survival at day 10, whereas hatchling exposure to cannibal tadpole cues caused a reduction in all these response variables (Table 5; Figure 5a–c). Tadpoles in control containers were 169.1 times more likely to survive to day 10 than tadpoles previously exposed as hatchlings to cannibal tadpole cues (odds ratio SE: 86.4, 331.1).

**TABLE 5:** Effect of early food subsidy (no, yes) and cannibal cues (no, yes) on subsequent tadpole growth (mass, mg), development (Gosner 1960 stage) and survival after 10 days (see Figure 5).

Response	Estimate ± SE	df	*F* / *χ* ^2^	*p*
Tadpole mass (mg)				
Food subsidy (yes)	−0.80 ± 2.31	1,32	0.001	0.9716
Cannibal tadpole cue (yes)	−36.05 ± 2.70	1,32	192.28	< 0.0001*
Food subsidy × cannibal tadpole cue		1,29	2.05	0.1630
Tadpole developmental stage				
Food subsidy (yes)	−0.25 ± 0.74	1,33	0.11	0.7438
Cannibal tadpole cue (yes)	−4.46 ± 0.23	1,33	413.94	< 0.0001*
Food subsidy × cannibal tadpole cue		1,29	4.47	0.0432*
Survival				
Food subsidy (yes)	0.92 (0.67, 1.27)	1	0.07	0.7976
Cannibal tadpole cue (yes)	0.006 (0.003, 0.011)	1	61.21	< 0.0001*
Food subsidy × cannibal tadpole cue		1	0.001	0.9734

*Note:* Interaction results are from the full interaction model. Main effects results are from separate analyses for each factor. Food supplement type (algae wafers vs. algae wafers + pond dirt) was retained as a covariate for models assessing tadpole mass (interaction effect: Food supplement type *p* = 0.046, food subsidy and cannibal cue main effects: Food supplement type *p* = 0.045), development stage (interaction effect: Food supplement type *p* = 0.0001, food subsidy main effect: Food supplement type *p* = 0.0463, cannibal cue main effect: Food supplement type *p* < 0.0001) and survival (interaction effect: Food supplement type *p* = 0.0001, food subsidy and cannibal cue main effects: Food supplement type *p* < 0.0001). *F* values are for mass and development stage results; chi‐squared value is for survival result. Effect size (Estimate) for survival is odds ratio with lower and upper SE values in parentheses.

*p < 0.05.

## 5 Discussion

Developmental plasticity is widespread in the early life stages of anurans, especially in response to cues of predation risk (Warkentin 1999, 2011a, 2011b; Gomez‐Mestre et al. 2006; Van Buskirk 2016; Schulte et al. 2023). However, the specific type of developmental plasticity exhibited by invasive cane toads in Australia, the accelerated development rate of pre‐feeding developmental stages in response to perceived risk of cannibalism (DeVore et al. 2021b; Crossland et al. 2024), is rare among amphibians. The only other records of such accelerated developmental responses are for salamander hatchlings (*Hynobius retardus*) responding to conspecific cannibal cues (Kishida et al. 2015) and frog eggs ( *Rana temporaria* ) responding to cues from a predatory planarian (Segev et al. 2015). Our results clarify several aspects of this cannibal‐induced developmental plasticity in invasive cane toads in Australia.

Firstly, the chemical that induces toad hatchling responses is located in the skin of cane toad tadpoles. Given that exposure of cane toad hatchlings to native Australian frog tadpoles does not induce negative carry‐over effects on growth, development or survival (our unpubl. data), the cue must be a chemical present in the skin of cane toad tadpoles but absent from the skin of native Australian tadpoles. One candidate is the bufonid toxin chemicals (bufadienolides) that are located in the skin of bufonid tadpoles (including cane toad tadpoles: Flier et al. 1980, Jara and Perotti 2009) but are absent from the skin of native Australian tadpoles because Australia lacks native bufonid anurans. This general concept of the scent of predator toxin functioning as a kairomone in amphibians is not unprecedented: larval California newts detect and avoid toxins (tetrodotoxin, TTX) that leach from the skin of cannibalistic adult newts into the surrounding water (Zimmer et al. 2006). Skin swabs of adult California newts also induce behavioural avoidance responses in conspecific larvae (Zimmer et al. 2006), just as skin swabs of cane toad tadpoles induced negative developmental plasticity in the present study.

However, whereas we have previously found that the maternally invested bufadienolides that induce cannibal attraction to hatchlings are also present in the parotoid secretions of adult cane toads (Crossland et al. 2012; Crossland et al. 2021; DeVore et al. 2021a, 2021b), the current study found no evidence that any of the compounds in adult toad parotoid secretions acted to induce a defensive response in hatchlings. Ontogenetic shifts across life stages in chemical profiles may contribute to the specificity of the cannibal tadpole cue. For example, although the tadpole and terrestrial life stages of cane toads share several bufadienolides (Hayes et al. 2009), the profile of some toxins, such as bufagenins, differs between these life stages (Kamalakkannan et al. 2017). Thus, the cue may be a chemical specific to the cane toad tadpole stage. Further work is required to identify the specific tadpole skin chemical(s) involved. This will likely require chemical and/or proteomics investigations to isolate and then purify or synthesise the candidate skin chemical(s), and then behavioural assays to test the chemical(s) in isolation. Such work has previously been done to identify the chemicals responsible for cannibalism attraction responses in cane toad tadpoles (Crossland et al. 2021).

Interestingly, rather than inducing negative responses on growth or development, hatchlings exposed to 1 mL adult parotoid gland secretions were significantly larger than controls at day 10 with a trend for faster development (*p* = 0.06). Several factors may account for this positive effect. For example, the parotoid secretions of adult common toads ( *Bufo bufo* ) contain proteins involved in cell metabolism and cell division (Kowalski et al. 2023); hence, cane toad hatchlings may have been exposed to such growth‐promoting proteins in cane toad parotoid secretions. More specifically, bufonid parotid gland secretions contain arginine (Chen and Kovaríková 1967), and exposure to arginine is known to promote growth in cane toad tadpoles (Crossland et al. 2019). Finally, the parotoid secretions we generated by squeezing adult toads would have contained mucus from skin mucus glands (Toledo and Jared 1995; Clarke 1997), and such mucus could potentially contain growth hormones that could be transmitted to developing hatchlings, as has been demonstrated for fish fry (Schütz and Barlow 1997). However, since cane toad hatchlings are non‐feeding, any of these mechanisms would require cane toad hatchlings to absorb the relevant parotoid secretion chemical(s) from solution.

Our experiments also demonstrated that the potency of toad tadpole skin swabs declined quickly once collected. Swabs collected 1 min prior to exposure to hatchlings reduced larval mass at day 10, whereas swabs collected 5 h or 24 h prior to hatchling exposure had no significant effect on day 10 mass. Skin swabs from all three time categories reduced developmental stage at day 10, but the effect was strongest for swabs collected 1 min prior to exposure. Interestingly, there was no effect of any skin swabs on survival at day 10. This latter result likely indicates that the swabbing process only collected a small amount of the total chemical cue present in tadpole skin because exposure to full‐sib live tadpoles during hatchling development in Experiment 2 did induce a significant reduction in survival at day 10, as has been previously demonstrated in this system (Crossland and Shine 2012; Clarke et al. 2015, 2016; McCann et al. 2019a, 2020; Crossland et al. 2024). Lower cue doses may impose fewer defensive costs, as defensive plastic responses can be dose‐dependent, with stronger cues inducing stronger defenses (McCoy et al. 2012; Rosenthal et al. 2023), and these costs have been found to be dose‐dependent in the cane toad cannibalism system (McCann et al. 2020).

The process of swabbing tadpoles and thereby removing some of the tadpole skin cue chemicals did not alter the potency of swabbed tadpoles to induce negative carry‐over effects on growth and development. Our gentle swabbing was unlikely to remove all chemical cue from tadpole skin, and so swabbed tadpoles retained sufficient chemical to cause negative effects on growth and development at day 10 comparable to un‐swabbed tadpoles. However, a surprising result was that cues from swabbed tadpoles had a greater negative effect on survival at day 10 than cues from un‐swabbed tadpoles. In our experiment, there was a 5 h interval between tadpoles being swabbed and hatchlings being exposed to these swabbed tadpoles. If the chemical responsible for inducing developmental plasticity is produced by cane toad tadpoles *de novo*, as is presumed, then during this 5 h interval the swabbed tadpoles may have altered chemical production to recover from swabbing, resulting in a difference in the quantity or diversity of skin chemical between swabbed versus un‐swabbed tadpoles at the time of hatchling exposure. For example, following depletion of bufadienolides located in the skin via hormonal treatment (norepinephrine), *Bufo bufo* tadpoles recover total bufadienolide quantity to control levels within 12 h via *de novo* synthesis; however, some of the individual bufadienolide compounds occur in higher concentrations after this recovery time (Tóth et al. 2019). The tadpole cues that induce developmental acceleration in cane toads have been hypothesised to be those that tadpoles produce as an anti‐predator defence (DeVore et al. 2021b), and tadpoles that have been exposed to predation cues have been found to induce stronger cannibal responses in hatchlings (Sarma et al. 2021). Thus, it is also possible that the swabbing process was perceived by tadpoles as a predator attack and that the swabbed tadpoles increased their cue production as a response. We also note that bufonid tadpole skin contains other compounds such as amines and peptides (e.g., Raices et al. 2020), and changes in the production of these chemicals following swabbing could also potentially alter stress‐related pathways in exposed hatchlings. It remains to be determined why swabbed tadpoles caused greater effects than un‐swabbed tadpoles on survival, but not on growth or development.

Our results also show that the developmental acceleration of cane toad hatchlings per se is not inherently costly. In contrast to cannibal tadpole cues, there was no carry‐over cost to temperature‐induced developmental acceleration; in fact, individuals that developed faster at higher temperatures also developed faster as tadpoles, with a tendency for an increased tadpole growth rate (*p* = 0.06). Similarly, Freidenburg (2017) found that temperature‐induced accelerated development of wood frog ( *Rana sylvatica* ) embryos caused no negative carry‐over effects on growth or development at 2 weeks post‐hatching.

Cannibal‐induced developmental acceleration in cane toad hatchlings has been found to occur as a trade‐off with hatchling growth, such that cannibal‐exposed individuals have reduced size when they transition into stage 25 feeding tadpoles. This size reduction at stage 25 predicts the magnitude of reduction in fitness during the subsequent tadpole stage (DeVore et al. 2021b; Crossland et al. 2024). In contrast, we found no evidence for a growth/development trade‐off for temperature‐induced developmental acceleration in cane toad hatchlings, with stage 25 tadpoles in low versus high temperature treatments being of similar size despite pre‐feeding development rates that were almost twice as fast at higher temperatures. Ultimately, there were no negative carry‐over effects for cane toad hatchlings exposed to temperature‐induced accelerated development. Similarly, Supekar and Gramapurohit (2024) found that size at stage 25 for the Indian skipper frog ( *Euphlyctis cyanophlyctis* ) is not affected by cues from dragonfly nymphs fed conspecific tadpoles during early development, and there was also no negative carry‐over effect on subsequent tadpole growth rate. Thus, the effect of developmental acceleration on size at tadpole stage 25 may underlie much of the costly carry‐over effects in cane toads. Consequently, if other mechanisms of developmental acceleration induce similar size reduction of stage 25 cane toad tadpoles, we would predict negative carry‐over effects on future fitness for these other mechanisms as well. However, any such effects may not be as strong as cannibal‐induced effects because the effects of cannibal cane toad tadpole cues are greater than those predicted by reduced growth effects alone, suggesting there are additional, unknown physiological costs associated with cannibal‐induced developmental acceleration for cane toads (Crossland et al. 2024).

One of the most striking results of our study was the inability of cane toad tadpoles to recover from early exposure to cannibal tadpole cues despite an abundance of food resources throughout development. This contrasts strongly with the ability of cane toad tadpoles to recover from earlier competitive effects of conspecific tadpoles on growth and development (McCann et al. 2019a; Crossland and Shine 2023) and also with the widespread ability of anuran larvae in general to recover from adverse conditions during early life once the stress is removed, including early nutritional stress (Audo et al. 1995; Capellán and Nicieza 2007; Squires et al. 2010; Orizaola et al. 2014; Pakkasama and Aikio 2003). Interestingly, the negative carry‐over effects of early exposure to cannibal tadpole cues affect both the growth and development of cane toad tadpoles, despite these two processes being capable of operating independently in anuran larvae (Wilbur and Collins 1973; Székely et al. 2017; Pintar and Resetarits Jr 2018). Our current study demonstrates that the reduction in tadpole growth/development trajectories induced by early exposure to cannibal cane toad tadpole cues is an example of a broader syndrome of developmental plasticity whereby phenotypic changes made during early ontogeny are irreversible regardless of changes in environmental conditions favorable for subsequent recovery (see also Taborsky 2017).

Our conclusions have direct implications for the role of environmental factors in affecting the magnitude of cannibal‐exposure costs. If the cannibal cue directly causes the costs, as we found, and those costs of reduced growth, development, and survival occur via a growth/development trade‐off during the hatchling stage and are irreversible, then variation in external conditions is likely to be largely irrelevant. For example, tadpole viability will be dramatically reduced by exposure to the cannibal cue under all nutrient conditions. Under the alternative but now rejected hypothesis that the impairment of growth, development and survival is a result of fast development per se, then factors such as low water temperature, which slows development, and abundant food, which allows fast growth, might ameliorate any impact of the cannibal cue. We conclude that such effects are unlikely. Consistent with this notion, McCann et al. (2020) found that the costs of hatchling exposure to cannibal tadpole cues for development rate to metamorphosis and survival to metamorphosis were equivalent for full‐sib cannibal‐exposed tadpoles raised in the laboratory at constant water temperature (30°C) versus raised in semi‐natural waterbodies that would have had significant diel variation in water temperature as well as higher nutrient availability. Hence, this mechanism likely operates throughout the cane toad's range within Australia for clutches that respond to cannibal cues by increasing development rate, regardless of locally available conditions of temperature and food supply within waterbodies used for spawning. The direct link between cannibal‐cue exposure and reduced larval viability strengthens the argument that this chemical cue might provide a new means of controlling invasive populations of cane toads in Australia (Clarke et al. 2015, 2016; McCann et al. 2019a, 2020).

## Author Contributions

**Michael R. Crossland:** conceptualization (equal), data curation (lead), formal analysis (equal), investigation (equal), methodology (equal), writing – original draft (equal). **Richard Shine:** conceptualization (equal), funding acquisition (lead), methodology (equal), resources (lead), supervision (lead), writing – original draft (equal). **Jayna L. DeVore:** conceptualization (equal), formal analysis (equal), investigation (equal), methodology (equal), writing – original draft (equal).

## Conflicts of Interest

The authors declare no conflicts of interest.

## Data Availability

The data and R script are available on figshare (https://doi.org/10.6084/m9.figshare.27115897).
